# The Influence of Early Life Nutrition on Epigenetic Regulatory Mechanisms of the Immune System

**DOI:** 10.3390/nu6114706

**Published:** 2014-10-28

**Authors:** Lorella Paparo, Margherita di Costanzo, Carmen di Scala, Linda Cosenza, Ludovica Leone, Rita Nocerino, Roberto Berni Canani

**Affiliations:** 1 Pediatric Food Allergy Unit, Department of Translational Medical Science, University of Naples Federico II, via S. Pansini 5, 80131 Naples, Italy; E-Mails: lorella.paparo@alice.it (L.P.); mara.dicostanzo@live.it (M.C.); carmen91ds@alice.it (C.S.); lindacosenza@libero.it (L.C.); ludovicaleone@hotmail.it (L.L.); ritanocerino@alice.it (R.N.); 2 European Laboratory for the Investigation of Food Induced Diseases, via S. Pansini 5, 80131 Naples, Italy; 3 CEINGE—Advanced Biotechnologies, University of Naples Federico II, via S. Pansini 5, 80131 Naples, Italy

**Keywords:** gut microbiota, short-chain fatty acids, butyrate, food allergy, pregnancy, children, DNA methylation, miRNA, histone deacetylase inhibitors

## Abstract

The immune system is exquisitely sensitive to environmental changes. Diet constitutes one of the major environmental factors that exerts a profound effect on immune system development and function. Epigenetics is the study of mitotically heritable, yet potentially reversible, molecular modifications to DNA and chromatin without alteration to the underlying DNA sequence. Nutriepigenomics is an emerging discipline examining the role of dietary influences on gene expression. There is increasing evidence that the epigenetic mechanisms that regulate gene expression during immune differentiation are directly affected by dietary factors or indirectly through modifications in gut microbiota induced by different dietary habits. Short-chain fatty acids, in particular butyrate, produced by selected bacteria stains within gut microbiota, are crucial players in this network.

## 1. Introduction

The immune system is exquisitely sensitive to environmental changes. One of the clearest reflections of this is the recent and dramatic rise in immune-mediated diseases, mainly allergies, with progressive modern urbanization.

Diet constitutes one of the major environmental factor that exerts a crucial effect on immune system development and function, and thus, it greatly influences many aspects of health and disease risk [1,2].

The rise in allergic diseases is fast becoming a major global health issue. While this was first evident in more developed countries of Australasia, Western Europe and North America, where more than 40% of the population may be affected at some stage [3,4], it is now also emerging in virtually all regions of the world undergoing industrial development and Westernization [5]. International trends provide some indication that environmental changes can affect immune function regardless of the genetic background. There is emerging evidence that the epigenetic mechanisms that regulate gene expression during immune differentiation are susceptible to these environmental influences [1]. Epigenetics is the study of mitotically heritable, yet potentially reversible, molecular modifications to DNA and chromatin without alteration to the underlying DNA sequence [6,7]. Increasingly, it is recognized that epigenetic marks provide a mechanistic link between environment, nutrition and disease. Though the DNA sequence is fairly permanent, epigenetic modifications are dynamic throughout the life course and can be heavily influenced by external factors [7]. The epigenetic program is encoded by specific histone modifications (methylation and acetylation) and DNA methylation patterns, which determine the degree of DNA compaction and, thus, the accessibility of genes for transcription. In addition to histone modifications and DNA methylation, there are also other gene regulatory networks, including microRNAs, small interfering RNAs and long non-coding RNAs, all of which serve to control gene expression [8]. Given that these modifications are reversible and sensitive to environmental factors, they provide a mechanistic link between environmental exposures, developmental programming and the risk for disease [9]. Early life nutrition may influence immune system development through direct epigenetic mechanisms. Some nutritional factors, like folate and vitamins B1, B2 and B12, can change DNA methylation [10]. Retinoic acid, garlic and dietary protein restriction may cause histone modification, and bioactive food compounds, like curcumin, genistein and retinoic acid, can decrease carcinogenic expression by miRNA [10]. Alternatively, dietary factors could influence epigenetic regulation of gene expression through an indirect mechanism mediated by a modulation of gut microbiota. The major metabolites produced by gut microbiota are short-chain fatty acids (SCFAs), such as butyrate, that have multiple beneficial effects at the intestinal and extra-intestinal level [11]. As more dietary fibers are ingested, SCFAs production increases [12]. The circulating SCFAs have a regulatory effect on macrophage and dendritic cell (DC) biology, supporting a strong connection between dietary fibers intake and immune response controlled by these cells [13]. Interestingly, the placenta has recently been shown to contain commensal microbes [14]. This suggests a potential role for maternal diet through an effect on placental commensal microbes on immune pathways during fetal development. Similarly, metabolites from gut microbiota, such as SCFAs, are present in breast milk, and this might be an important point of interaction between metabolites and the immune system [15,16,17,18].

## 2. Nutritional Epigenetic Effects on Early Development

Maternal nutrition plays an essential role in offspring health and development. Critical stages include: preconception, affecting oocyte development and uterine environment preparation, gestation, affecting uterine environment and placental nutrient transfer, and postnatal, through lactation [19,20,21]. It remains debatable which stage is most important, but arguably the most complex cellular events occur during gestation. During this time, embryo development requires a well-orchestrated and tightly-regulated cascade of genetic, molecular and biochemical events. New data reinforce the importance of *in utero* exposures to nutrients in fetal immune development and in programming the susceptibility to allergic diseases [22]. Prenatal environmental exposures may have important and permanent effects on epigenetic mechanisms. Nutriepigenomics is an emerging discipline examining the role of dietary influences on gene expression. Ultimately, DNA methylation events and dietary practices, particularly micronutrient intake, may influence disease phenotypes [23] (Table 1). Methylation occurs after replication and almost exclusively affects position five of the pyrimidine ring of cytosines in the context of the dinucleotide sequence, CpG [24]. Approximately 75% of mammalian CpG dinucleotides are methylated. Clusters enriched in CpGs (CpG islands) are found in locus control regions, promoter regions and the first exons of many genes. Throughout the genome, CpG islands are generally much less methylated, potentially allowing transcription [25]. The methyl groups needed for all biological methylation reactions are derived from methyl donors and from cofactors carrying one-carbon units. A key pathway of these reactions is the metabolic cycling of methionine [26]. Novel experiments investigating maternal methyl donor supplementation *in utero* clearly demonstrated the impact of early nutrition epigenome shaping. Hypermethylation of a gene crucial in lymphocyte regulation, *Runx3*, arising in offspring exposed to methyl donors *in utero* has been considered to be involved in increased allergic airway disease development and severity [27]. Furthermore, C57BL/6 mice exposed to methyl donor supplementation *in utero* exhibited enhanced colitis susceptibility, which was also associated with aberrant DNA methylation among genes associated with immunologic processes [28]. Dietary folate is the most extensively studied micronutrient in animal and epidemiological DNA methylation research. Folate is reduced to dihydrofolate (DHF) and subsequently to tetrahydrofolate (THF), serving as a single carbon donor in the form of 5-methyl THF. Consequently, 5-methyl THF feeds into the one-carbon metabolism cycle by donating its methyl group to homocysteine, converting it to methionine. Cofactor B vitamins provide the enzymatic support necessary for these transformations, making it possible for dietary folate to feed into the one-carbon metabolism cycle to replenish cellular S-adenosyl-methionine (SAM). For this reason, folate supplementation has generally been associated with increased DNA methylation and *vice versa* for folate restriction [25]. A study on rats found that normal dietary folate levels with supplemental selenium yielded greater methylation of DNA in colon and liver than when diets contained only one or neither of the two nutrients [29]. Another study investigated the effect of folate deficiency during pregnancy and lactation and of high-fat feeding from weaning on DNA methylation and the expression of selected genes in adult offspring brain in a mouse model. The results suggest that low folate supply during early life may cause an epigenetic effect in offspring, leading to an increased sensibility to further dietary insults [30].

Human studies have also investigated the role of maternal folate status and offspring methylation. A cross-sectional study of pregnant women at the time of delivery looked at the imprinted gene *Igf2* [31]. Cord blood and maternal blood were collected at time of delivery, and serum folate concentrations were determined for both specimens [32]. Methylation-specific PCR determined that maternal and cord blood folate concentrations were not associated with methylation outcomes in the P2 and P3 promoters of *Igf2*. Additionally, hypomethylation within the *Igf2* promoter of umbilical cord blood leukocytes was inversely associated with maternal report of folate supplementation during pregnancy [33]. Maternal plasma folate and homocysteine levels and/or polymorphisms in folate metabolizing genes have been implicated in inorganic arsenic (iAs) metabolism, a common, naturally occurring drinking water contaminant, and in susceptibility to iAs toxicity [34,35]. Tsang *et al*. showed that *in utero* exposure of mice to iAs, combined with high gestational folate intake, results in low fetal weight at gestation Day 18, dramatic changes in global DNA methylation in fetal liver and in aberrant CpG island methylation of genes associated with fetal development [36]. In another study, Schaible *et al*. provided evidence that maternal methyl-donors (MDs) supplementation increases offspring colitis susceptibility, which is associated with persistent epigenetic and prolonged microbiomic changes. These findings underscore that epigenomic reprogramming relevant to mammalian colitis can occur during early development in response to maternal dietary modifications and suggest that the prenatal reprogramming of mucosal immunology upon maternal MDs supplementation creates a persistent effect on the enteral microbiome by inducing a longstanding modification of its physiologic development [28]. Another study examined the effect of methyl donor-restricted (MR) and methyl donor-supplemented (MS) diets on the expression of methylation-sensitive T-cell genes and lupus disease, using a mouse model. The authors found that MS diet reduced anti-dsDNA antibody to near background levels in transgenic mice with defective Erk-triggered lupus-like disease, suggesting that micronutrients that enhance transmethylation reactions may ameliorate lupus disease via epigenetic mechanisms. Similarly, the reduced methionine content of the MR diet, together with reduced Erk pathway activity, could have exacerbated lupus disease via epigenetic mechanisms by causing DNA hypomethylation and enhanced immune gene expression [37]. Choline and betaine are indirect methyl group donors for one-carbon metabolism. Choline deficiency during gestational Days 12–17 decreased methylation within a CpG site located in the *Calb1* promoter of the fetal hippocampus [38]. Wang *et al*. identified a new direct link between the gut microbiota-dependent metabolism of dietary phosphatidylcholine and cardiovascular disease pathogenesis, which represents a leading cause of death and morbidity worldwide. These results suggest that an appropriately-designed probiotic intervention may serve as a preventive and therapeutic strategy for these conditions [39]. Hypermethylation at the DMR 2 of *Igf2* in rat liver, following exposure to a choline-deficient diet from embryonic Days 11–17, was correlated with hypomethylation of CpG sites within *Dnmt1* (DNA methyltransferase 1), the gene encoding DNA methyltransferase, and these results suggest that early choline-deficiency is able to deprive DNA methylation machinery of proper substrates of one-carbon metabolism, leading to increased expression of methyltransferases [40]. Vitamins B6 and B12 are cofactors involved in the regulation of the catalytic activity of enzymes from the folate cycle, thus determining SAM bioavailability. Supplementing diets with these vitamins will contribute to the maintenance or establishment of DNA methyl marks [2]. High ethanol consumption inhibits the availability of vitamins B6 and B12, thus interfering with the production of SAM and appropriate DNA methylation, through the folate/methionine cycles [41].

Ba *et al*. assessed vitamin B12 and folate status in pregnant women at the time of birth. Cord blood *Igf2* methylation at the P3 promoter was inversely correlated with the maternal serum level of vitamin B12. Additionally, maternal blood methylation in the P2 promoter was inversely correlated with maternal serum vitamin B12 level [32]. A wide range of nutrients, including fat feeding, protein, alcohol, vitamin E, hormones and a number of polyphenols, may alter expression of specific miRNAs [42,43,44,45]. Recent studies show that maternal high fat feeding during gestation and lactation changed the expression of 23 miRNAs in liver in the offspring [46]. Keller *et al*. showed that a large set of miRNAs in the skeletal muscle of obese Zucker rats is responsive to carnitine supplementation, suggesting a novel mechanism through which carnitine exerts its multiple effects on gene expression [47]. Vitamin E deficiency in rats caused a downregulation of miR-122a and miR-125b, which contribute to regulating lipid metabolism and inflammation, respectively [48]. Wang *et al*. observed that maternal exposure to ethanol also changed the expression of several miRNAs in the fetal brain from the offspring [49]. There are also some studies linking macro-nutrition (e.g. carbohydrates) to transgenerational epigenetic changes and immune disease. Miao *et al*. [50] reported that after chronic exposure of the human monocytic cell line, THP-1, to high glucose, histone 3 acetylation at lysine 9 and lysine 14 was increased at the TNF-α and COX-2 promoters. Another study showed that transient exposure to hyperglycemia induced long-lasting activating epigenetic changes in the promoter of the nuclear factor kB subunit p65 in aortic endothelial cells, both *in vitro* and in non-diabetic mice. They found that an increase of monomethylation of histone 3 lysine 4 by the histone methyltransferase Set7, in the proximal promoter region of p65, caused an upregulation of p65 gene expression, leading to a sustained increase in the expression of the NF-κB-responsive proatherogenic genes, MCP-1 and VCAM-1 [51]. These epigenetic changes persist for at six days and may be responsible for the persistent atherogenic effects during subsequent normoglycemia [51]. Recent findings showed that the hyperglycemic intrauterine environment of gestational diabetes mellitus (GDM) results in a high risk of diabetes in offspring by altering epigenetic modification. In addition to intergenerational transmission (F1 offspring), intrauterine hyperglycemia may also have effects on the second generation (F2 offspring) [52]. Using a GDM mouse model, the authors demonstrated that the expression of imprinted genes *Igf2* and *H19*, in both F1 and F2 offspring, was downregulated in pancreatic islets, caused by abnormal methylation status of the differentially methylated region, which may be one of the mechanisms for impaired islet ultrastructure and function. Altered *Igf2* and *H19* gene expression was also found in sperm of adult F1-GDM with or without impaired glucose tolerance, indicating that epigenetic changes in germ cells contributed to transgenerational transmission [52].

**Table 1:** Main nutritional factors influencing the immune system through an epigenetic mechanism.

Nutritional Factors	Epigenetic Mechanism
Folic acid	DNA methylation [25,29,30,31,32,33,34,35,36]
Choline and betaine	DNA methylation [38,39,40]
Vitamins	DNA methylation [2,32] microRNAs [48]
Dietary fibers (butyrate production by gut microbiota) [12]	DNA methylation [21] Histone modifications [10,53]
Fat feeding, protein, hormones	microRNAs [42,43,44,45]
Ethanol	DNA methylation [41] microRNAs [42,43,44,45,49]
Carbohydrates	DNA methylation [50,51,52]

## 3. Gut Microbiota as a Nutriepigenomic Player

**Figure 1:**
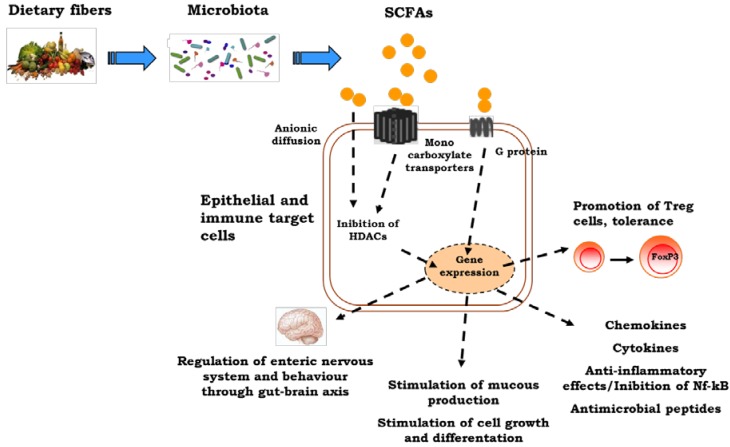
The influences of early nutrition on the immune system. Dietary factors may induce direct epigenetic modifications and/or may influence epigenetic mechanisms through a modulation of gut microbiota composition and function. The short-chain fatty acid, butyrate, produced by gut microbiota exerts a broad range of epigenetic effects influencing immune system development and function. SCFAs, short-chain fatty acids.

Gut microbiota plays a critical role in the establishment and maintenance of body health (Figure 1). Commensal bacteria are involved in the fermentation of dietary fibers in the colon, leading to SCFAs production. Among the SCFAs, butyrate has received particular attention for its multiple beneficial effects from the intestinal tract to peripheral tissues [10]. Butyrate-producing bacteria represent a functional group, rather than a coherent phylogenetic group. Numerically, two of the most important groups of butyrate producers appear to be *Faecalibacterium* *prausnitzii*, which belongs to the *Clostridium leptum* (or clostridial cluster IV) cluster, and *Eubacterium rectale/Roseburia* spp., which belong to the *Clostridium coccoides* (or clostridial cluster XIVa) cluster of Firmicutes bacteria [54]. Studies have highlighted the profound effect of diet on gut microbiota composition and the connection to immune pathways [55,56,57]. Inadequate colonization of infant gut or alteration in the microbiota profile (dysbiosis) is now considered a strong risk factor for chronic disorders, such as allergic and autoimmune diseases [58,59,60]. A recent study found a significant relationship between gut microbiota constituents at one month of age and the later development of specific serum IgE against food proteins. In this study, 952 participants of the Child, Parent and Health: Lifestyle and Genetic Constitution (KOALA) Birth Cohort Study provided a stool sample at one month of age and were characterized by cow’s milk, egg and peanut food-specific IgE measurement at 1, 2 and 67 months of age. *Clostridium* *difficile* was more often found in the fecal samples of subjects with a family history of allergic disease sensitized to food later in childhood [54]. Restoring the microbiota profile may be effective in the prevention or treatment of allergic and inflammatory diseases. These observations have led to the idea that probiotics, which have the potential to restore the intestinal microbiota balance, may be effective in preventing the development of chronic immune-mediated diseases [55]. Ghadimi *et al*. investigated the effects of two probiotics, *Bifidobacterium breve* (DSMZ 20213) and *Lactobacillus rhamnosus GG* (*LGG*) (ATCC 53103), in an *in vitro* model of the intestinal mucosal immune system. The results of this study showed that probiotics can inhibit translation of IL-23, IL-17 and CD40 genes by epigenetic process involving reducing histone acetylation and enhancing DNA methylation [61]. A recent study showed that intestinal microbiota of pregnant women can play a very important role in epigenetic activation or suppression of gene expression in the pregnancy period, because SCFAs produced by indigenous gut and vaginal microbiota of pregnant women can penetrate via placenta into fetus and affect body composition in the natal and postnatal periods of life [17]. Therefore, diet or probiotic supplementation may induce an alteration of maternal indigenous microbial and long-term consequences in offspring by epigenetics mechanism that occur during embryonic and fetal development [39].

## 4. The Positive Role of Butyrate Epigenetic Effects on Children’s Health

Butyrate has a pivotal role in the context of “gut/body health”. Its production is dependent on diet and intestinal microbiota composition. Butyrate is also able to modulate intestinal microbiota through regulation of lumen pH and to exert many beneficial extraintestinal effects through epigenetic mechanisms [62].

Butyrate exerts a general anti-inflammatory effect [63]. Mechanisms for butyrate anti-inflammatory functions are at least in part mediated through histone deacetylase (HDAC) inhibition and activation of metabolite-sensing G-protein-coupled receptors (GPCRs). GPCR signaling is a common pathway for K^+^ efflux or Ca^2+^ flux, and it is possible that this is central to the activation of the NALP3 inflammasome and production of IL-18 [64]. Most metabolite-sensing GPCRs and their ligands (including GPR43 and SCFAs, GPR120 and omega-3 fatty acids, GPR109A and butyrate and nicotinic acid and possibly GPR35 and kynurenic acid and pamoic acid) facilitate anti-inflammatory effects [65]. GPR43 (also called free fatty acid receptor 2 or *Ffar2*) and GPR109A appear to be important for gut homeostasis, and both are expressed by the colonic epithelium, by leukocytes (such as neutrophils and macrophages) and by Treg cells [66,67].

Lack of GPR109A, a metabolite sensor for butyrate, is associated with fewer colonic Treg cells as a result of the reduced ability of colonic macrophages and DCs to promote their development [67]. GPR43-deficient (*Ffar2^−/−^*) mice show exacerbated inflammation in models of airway hypersensitivity, DSS-induced colitis and rheumatoid arthritis when treated with SCFAs [65]. Smith *et al*. observed that SCFA treatment of *Ffar2^−/−^* mice and *Ffar2^+/+^* littermates enhances Treg cell frequency and number in *Ffar2^+/+^*, but not for *Ffar2^−/−^* mice, suggesting that SCFA mediated enhanced Treg cells’ suppressive capacity was also dependent on *Ffar2.* They show also that SCFA treatment of *Ffar2^+/+^* mice reduced Treg cells’ HDAC6 and HDAC9 expression and enhanced histone acetylation. These results suggest that SCFAs via *Ffar2* may affect Treg cells through HDAC inhibition [67]. SCFAs are absorbed as anionic forms or actively transported via monocarboxyl transporters (MCT1 and SMCT1) into colonocytes. Intracellular SCFAs regulate HDACs with respect to changes in gene expression and signal transduction. HDAC inhibition mediates the regulatory activities of SCFAs in cellular processes, such as the induction of antimicrobial peptides, mucins, trefoil factors (TFF), chemokines and cytokines, which together promote gut immunity and mediate intestinal inflammation [68]. Hamer *et al*. provided evidence that butyrate is able to suppress nuclear factor-B (Nf-κB) activation and interferon γ production and to upregulate peroxisome proliferator-activated receptor γ (PPARγ), through the inhibition of HDAC [53]. In ulcerative colitis (UC), butyrate metabolism is impaired due to a defect in the butyrate oxidation pathway and/or transport [69]. A defect in butyrate uptake deriving from reduced butyrate carrier MCT1 (also called *SLC16A1*) mRNA levels at the gut level has been demonstrated in patients with UC [70]. Santhanam *et al*. described a defect in the mitochondrial enzyme, acetoacetyl CoA thiolase (encoded by the gene *ACAT2*), in UC patients. This enzyme catalyzed the last step of butyrate oxidation and was significantly impaired in UC patients and unrelated to disease severity [71]. The mRNA expression of the other genes involved in the butyrate oxidation pathway was also downregulated in inflammatory disease [69].

Evidence is also emerging of microbial-derived molecules with neuroactive functions that can have influence across the brain-gut axis. For example, γ-aminobutyric acid, serotonin, catecholamines and acetylcholine may modulate neural signaling within the enteric nervous system, when released in the intestinal lumen, and, consequently, brain function [72]. Early exposure to gut microbiota is essential for the postnatal development of the enteric nervous system (ENS) [73]. In germ-free mice (GF), Collins *et al*. demonstrated that the myenteric plexus of the jejunum and ileum was abnormally patterned with an overall decrease in nerve density when compared with equivalent segments of small intestine from specific pathogen-free (SPF) colonized animals. Myenteric ganglia in GF mice, furthermore, contained fewer neuronal cell bodies than those found in SPF-colonized small intestine with an increase in the proportion of inhibitory nitrergic neurons [73]. In a double-blind, placebo-controlled, multicenter trial, 51 patients with active distal ulcerative colitis (UC) were treated with rectal enemas containing either 5-aminosalicylic acid (5-ASA) or 5-ASA plus sodium butyrate. The combined treatment with topical 5-ASA plus sodium butyrate resulted in a significant improvement of the disease activity score compared to that observed in patients treated with 5-ASA alone [74]. Butyrate suppress the major TH1-skewing factors, IL-12 and IFN-γ, which influence the development of a subsequent T-cell response, and it increases the secretion of anti-inflammatory cytokines IL-10. However, this effect is not confirmed in all studies [75,76,77]. In addition, Usami *et al*. reported a reduced LPS-induced TNF-α secretion by 0.5 mM butyrate, indicating a therapeutic potential of butyrate by intravenous or oral administration [78]. An imbalance in gut microbiota composition has been also associated with food allergies. Recently, Nakayama *et al*. profiled the fecal bacteria compositions in allergic and non-allergic infants by using the 16S rRNA gene short tag pyrosequencing approach and correlated some anomalies in the microbiota with allergy development in later years [79]. CD4+ Treg cells, which express the *Foxp3* transcription factor, play a critical role in the maintenance of immune homeostasis and oral tolerance. Atarashi *et al*. [80] showed that in mice, the spore-forming component of indigenous intestinal microbiota, particularly clusters IV and XIVa of the genus, *Clostridium*, major butyrate producers, promotes Treg cell accumulation in the colonic mucosa. Our study showed that treatment of cow’s milk allergy (CMA) infants with an extensively hydrolyzed casein formula (eHCF) supplemented with the probiotic, *LGG*, accelerates oral tolerance acquisition to cow’s milk [81,82]. We tested also the hypothesis that eHCF plus *LGG* induced an effect on oral tolerance thanks to the influence of this dietary intervention on the composition of the gut microbiota [83]. Treatment with eHCF plus *LGG* expanded gut microbiota populations in newly diagnosed CMA infants, was associated with immunoregulatory effects and significantly increased butyrate production at the intestinal level. The protective effects of butyrate were also explored in a mouse model of CMA, where oral butyrate treatment alleviates the allergic response in β-lactoglobulin-sensitized mice [84]. These findings suggest a potential innovative therapeutic approach for infants affected by CMA, based on the effect of bacterial metabolites on host immunity and human health.

## 5. Conclusions

Nutriepigenomics is an emerging discipline examining the role of dietary influences on gene expression. An increasing amount of evidence suggests the pivotal role of epigenetic mechanisms in many positive effects elicited by nutrients and by diet-induced regulation of gut microbiota composition and function. The potential of these epigenetic mechanisms may represent innovative targets for new preventive and therapeutic strategies for a wide number of immune-mediated diseases.
